# Performance of the time-resolved ultra-small-angle X-ray scattering beamline with the Extremely Brilliant Source

**DOI:** 10.1107/S1600576721012693

**Published:** 2022-02-01

**Authors:** Theyencheri Narayanan, Michael Sztucki, Thomas Zinn, Jérôme Kieffer, Alejandro Homs-Puron, Jacques Gorini, Pierre Van Vaerenbergh, Peter Boesecke

**Affiliations:** a ESRF – The European Synchrotron, 38043 Grenoble, France

**Keywords:** small-angle X-ray scattering, SAXS, ultra-small-angle X-ray scattering, USAXS, X-ray photon correlation spectroscopy, XPCS, time-resolved studies

## Abstract

The new technical features and enhanced performance of the ID02 beamline with the Extremely Brilliant Source (EBS) at the ESRF are described. The beamline enables static and kinetic investigations of a broad range of systems from ångström to micrometre size scales and down to the sub-millisecond time range by combining different small-angle X-ray scattering techniques in a single instrument. In addition, a nearly coherent beam obtained in the high-resolution mode allows multispeckle X-ray photon correlation spectroscopy measurements down to the microsecond range over the ultra-small- and small-angle regions. While the scattering vector (of magnitude *q*) range covered is the same as before, 0.001 ≤ *q* ≤ 50 nm^−1^ for an X-ray wavelength of 1 Å, the EBS permits relaxation of the collimation conditions, thereby obtaining a higher flux throughput and lower background. In particular, a coherent photon flux in excess of 10^12^ photons s^−1^ can be routinely obtained, allowing dynamic studies of relatively dilute samples. The enhanced beam properties are complemented by advanced pixel-array detectors and high-throughput data reduction pipelines. All these developments together open new opportunities for structural, dynamic and kinetic investigations of out-of-equilibrium soft matter and biophysical systems.

## Abstract 2

The new technical features and improved performance of the time-resolved ultra-small-angle X-ray scattering beamline at the ESRF are presented. The beamline enables static and time-resolved investigations from ångström to micrometre size scales down to the sub-millisecond time range and coherent scattering studies in the ultra-small-angle region. Among the main applications are the elucidation of static and transient hierarchical structures in soft matter and biophysical systems, and the dynamics of out-of-equilibrium complex fluids.

## 1. Introduction

Recently, the new generation of synchrotrons based on multi-bend achromat storage-ring lattices have come into operation (Eriksson *et al.*, 2014 ▸; Raimondi, 2016 ▸; Rodrigues *et al.*, 2019 ▸). These sources have enhanced the brightness and transverse coherence of X-ray beams by more than an order of magnitude compared with third-generation storage rings (Weckert, 2015 ▸). The increased brilliance and coherence are both very attractive for scattering methods such as small-angle X-ray scattering (SAXS) and X-ray photon correlation spectroscopy (XPCS). Challenges remain in exploiting these outstanding source properties to address contemporary issues in a broad range of scientific disciplines. In particular, the high brightness comes with the baggage of correspondingly increased radiation damage, at least in the case of synthetic soft materials and biological specimens. This article deals with the performance of SAXS and related techniques at the recently opened Extremely Brilliant Source (EBS) at the ESRF (Raimondi, 2016 ▸). The EBS is based on the hybrid multi-bend achromat concept, operating at an electron energy of 6.0 GeV with operational root-mean-square (r.m.s.) horizontal and vertical emittances of 110 pm rad and 5 pm rad, respectively.

High-brilliance SAXS, and related techniques such as wide-angle X-ray scattering (WAXS) and ultra-small-angle X-ray scattering (USAXS), have been widely used in the investigation of soft matter and noncrystalline biological systems (Narayanan & Konovalov, 2020 ▸; Jeffries *et al.*, 2021 ▸). These scattering methods offer high reciprocal-space and temporal resolutions simultaneously, which makes them suitable for investigations of transient processes. Different combinations of scattering experiments with other thermophysical, rheological and biophysical methods further enhance the information which can be derived (Narayanan & Konovalov, 2020 ▸; Sakurai, 2017 ▸; Jeffries *et al.*, 2021 ▸). Moreover, the high degree of coherence enables probing of the equilibrium dynamics using XPCS for appropriate systems (Lehmkühler *et al.*, 2021 ▸). The availability of high frame rate pixel-array detectors has broadened the scope of multispeckle XPCS, in particular for probing fast dynamics down to the microsecond range (Zhang *et al.*, 2018 ▸; Zinn *et al.*, 2018 ▸). Despite the remarkable progress in microscopy, USAXS continues to be a useful technique for the investigation of optically opaque and polydisperse systems including *in situ* processes (Ilavsky *et al.*, 2018 ▸; Narayanan *et al.*, 2018 ▸; Pauw *et al.*, 2021 ▸). The high degrees of coherence and collimation are beneficial for performing USAXS in the pinhole geometry using a two-dimensional detector. Pinhole two-dimensional USAXS is very informative for the investigation of highly oriented specimens and kinetic processes (Kishimoto *et al.*, 2014 ▸; Narayanan *et al.*, 2018 ▸; Chumakov *et al.*, 2019 ▸).

Beamline ID02 at the ESRF is a multipurpose SAXS/USAXS/WAXS and XPCS instrument. The technical features of the instrument cover a scattering vector range of 0.002 ≤ *q* ≤ 50 nm^−1^ typically with two sample-to-detector distances and a single beam setting for an X-ray wavelength (λ) of 1 Å [*q* is the magnitude of the scattering vector given by 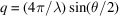 , with θ the scattering angle]. This broad *q* range spans in excess of four orders of magnitude in reciprocal-space dimension and, together with sub-millisecond time resolution, enables a wide range of dynamic studies from the molecular scale to the upper limit of colloidal dimensions. In the high-resolution mode, two-dimensional USAXS patterns down to *q* < 0.001 nm^−1^ can be recorded, and multispeckle ultra-small-angle XPCS (UA-XPCS) measurements can be performed for suitable systems (Zinn *et al.*, 2020 ▸). The main applications of the instrument include the elucidation of static and transient hierarchical structures, and nonequilibrium dynamics in soft matter and biophysical systems.

This article presents the main improvements in the performance of the beamline with the EBS upgrade. A direct consequence is that the new source enables relaxation of the collimation conditions for the high-brilliance mode (2–6 × 10^13^ photons s^−1^) covering 0.002 ≤ *q* ≤ 50 nm^−1^, resulting in a lower parasitic background. The high-resolution and coherence modes require tighter collimation but still with flux in excess of 10^12^ photons s^−1^.

## 2. Principal beamline components

**Figure 1:**
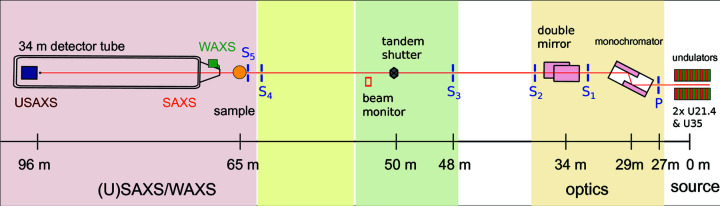
The schematic layout of the TRUSAXS (ID02) beamline, indicating the locations of the main components. Colours represent different sections of the beamline.

Fig. 1 ▸ schematically displays the layout of the beamline. The layout as such is unchanged from the previous publications (Van Vaerenbergh *et al.*, 2016 ▸; Narayanan *et al.*, 2018 ▸; Sztucki *et al.*, 2019 ▸) but the performances of certain critical components have been significantly improved. The beamline consists of the undulators, cryogenically cooled monochromator, focusing optics, collimation slits and sample environments, and the detector tube and different detectors installed in it. With the EBS, three well separated slits (P, S_3_ and S_5_) are generally sufficient to obtain the desired collimation, thereby significantly curtailing the parasitic background in the ultra-small-angle region. The SAXS/USAXS/XPCS detectors are enclosed within the wagon inside the detector tube and the WAXS detector is installed outside the entrance cone of the tube in air.

### 2.1. Undulator source

The EBS electron-beam properties are similar for all straight sections, with nominal FWHM size and divergence of 9 µm and 3.3 µrad vertically and 70 µm and 10 µrad horizontally, respectively. In addition, the electron-beam energy (6.0 GeV) is very close to that of the previous storage ring. As a result, the undulators, two phased U21.4 and a single U35 of length 1.6 m each and minimum gap of 11 mm, remain unchanged. Nevertheless, a future upgrade to a longer device of 2.3 m, with a minimum gap of 9 mm and a shorter period of *ca* 20 mm, is foreseen.

At present, the U21.4 undulators are primarily used for fixed-energy (*E*) operations (at 12.23 keV and up to a maximum of 15 keV), and the U35 device covers the full energy range of the beamline, 8–20 keV. Due to the phasing of the U21.4 undulators, the flux increases by about a factor of 2.5–3 compared with two separate devices when the primary slits are closed to 0.4 × 0.4 mm.

**Figure 2:**
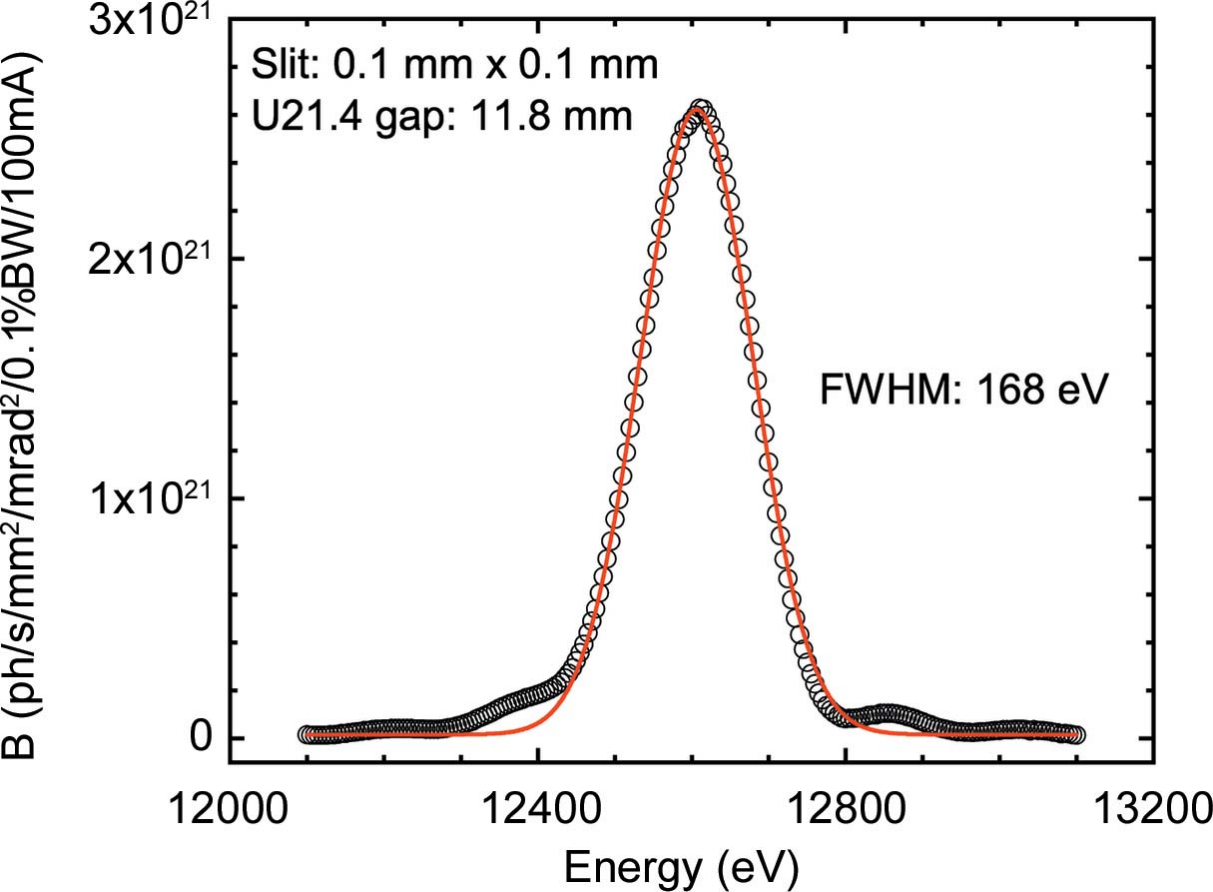
The measured brilliance of the undulator (U21.4) beam after the monochromator, with the primary slits closed to 0.1 × 0.1 mm. The continuous line is a fit to a Gaussian profile with an FWHM of 168 eV.

Fig. 2 ▸ displays the measured brilliance (*B*) of the central U21.4 device. An important feature of the EBS is the symmetry of the spectrum with relatively narrow bandwidth (BW). The combination of two U21.4 undulators and a primary slit aperture of 0.8 × 0.8 mm provides a monochromatic (Δ*E*/*E* ≃ 2 × 10^−4^) flux of about 10^14^ photons s^−1^ at the sample position. Finally, a polished diamond window (thickness ∼300 µm) separates the vacuum of the front end from the beamline optics.

### 2.2. Beam optics

The principal optical elements comprise the primary and secondary slits, the liquid nitrogen cooled monochromator and the focusing optics. The monochromator is an ESRF generic design, which was refurbished to improve the heat-load capacity and preservation of the beam spectral properties. The channel-cut Si 111 crystal (in the form of a *Z*-cut that allowed finer polishing of the two diffracting surfaces) was fabricated in-house. The channel width of 5 mm results in an increase in the beam height from the polychromatic beam by 9.9 mm at 12.23 keV, and this height offset is required for an efficient blocking of the bremsstrahlung radiation around the primary beam. The height offset varies by about 50 µm when the energy is increased from 12.23 to 16 keV. The improvement of the cooling circuit now enables typical heat load and power density in excess of 200 W and 100 W mm^−2^, respectively, without a significant loss in intensity and broadening of the rocking curve (Zhang *et al.*, 2013 ▸). The monochromator is calibrated with seven different absorption edges between 7 and 24 keV. The reproducibility of the crystal rotation stage is better than 0.3 eV.

The focusing optics part remains unchanged from that described before (Narayanan *et al.*, 2018 ▸), involving a double-mirror setup that allows sagittal and meridional focusing along the vertical and horizontal directions, respectively. This scheme has the advantage that the vertical focused beam parameters are less sensitive to the sagittal slope error of the mirror, essentially preserving the ideal source properties. However, the improved horizontal source properties make the horizontal focusing properties more sensitive to the meridional slope error. Nevertheless, improvements in the horizontal source properties already make the horizontal beam size smaller by a factor of 5 and the divergence is reduced by 50%. A further improvement in the horizontal and vertical beam sizes is feasible by introducing a secondary focusing element based on compound refractive lenses. This option has not been pursued yet due to the consideration of radiation damage, and a 20 µm beam spot on the sample is already obtained by a combination of focusing and slit collimation, albeit with a reduced flux of about 10^12^ photons s^−1^. The beam intensities are measured by means of monitors based on a Kapton foil (25 µm) scatterer and a silicon p-i-n photodiode (Hamamatsu Photonics). These intensity monitors (*I*_0_) are calibrated using a reference photodiode where the absolute photocurrent and absorption are measured directly.

**Figure 3:**
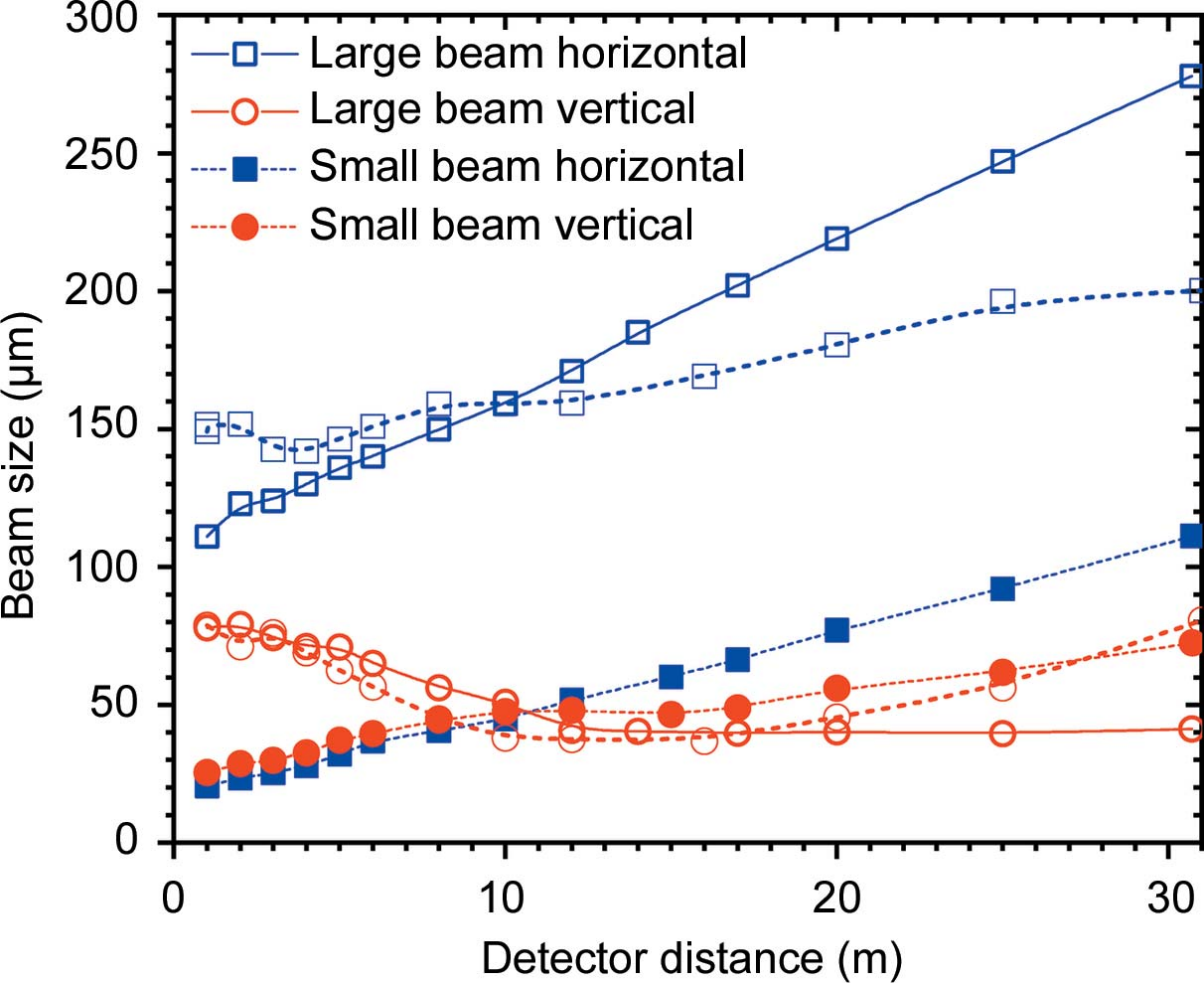
Measured FWHM vertical and horizontal beam sizes along the detector tube for different inclinations of the double-mirror setup. The large-beam values correspond to inclinations of 3.2 mrad (open symbols with dashed lines) and 2.7 mrad (open symbols with continuous lines). The inclination for the small beam is 3.8 mrad (filled symbols).

The beam focus is optimized between the sample position and farthest detector position by varying the inclination of the double-mirror assembly from about 3.8 to 2.6 mrad while maintaining the reflected beam along the same path. Changing the inclination of a toroidal mirror has the disadvantage that the horizontal and vertical focal lengths change in opposite directions. However, for most practical purposes, it is sufficient to optimize the beam size for one particular distance, and the horizontal beam size can be further tuned using slits S_3_ and S_4_. In the standard configuration (flux ≃ 2 × 10^13^ photons s^−1^), the minimum FWHM vertical beam size varies from approximately 30 to 60 µm from the sample to the farthest detector position and the corresponding horizontal beam size changes from about 120 to 280 µm (for a primary slit size of 0.4 × 0.4 mm). Fig. 3 ▸ depicts the beam size measured along the detector tube for two different mirror inclinations using a high-resolution camera (beam viewer). For the large-beam case (flux ≃ 2 × 10^13^ photons s^−1^), the vertical beam size is optimized at either the farthest or an intermediate detector position. The latter provides a lower beam divergence, and therefore a higher *q* resolution (Δ*q*) and lower parasitic background. In the case of a small beam, the beam size is first minimized by the mirror at the sample position and then slit collimated to a beam spot of about 20 µm on the sample but with a significantly reduced flux (∼10^12^ photons s^−1^). The horizontal beam size typically increases as a function of sample-to-detector distance, with maximum divergences of about 5 and 2 µrad for small and large beams, respectively. Vertically, the size and divergence of the beam are mainly adjusted by the focusing mirror as smaller slit sizes (<60 µm) broaden the beam due to diffraction. Enlargement of the beam from the ideal source size is attributed to optical magnification and residual optical aberrations. In the standard configuration, the horizontal and vertical beam sizes are optimized at the middle of the detector tube for a magnification factor of ∼1.4.

The horizontal beam divergence could be further reduced using the multiple-bounce pseudo-channel-cut crystals based collimation system (Sztucki *et al.*, 2019 ▸) but with significant reduction in the flux. For variable beam sizes and coherence applications, the slit collimation is sufficient. In the standard high-flux configuration, the beam is collimated using the high-power primary slits (P) and secondary slits (S_3_). In that case, S_4_ is unused and S_5_, comprising the scatterless slits (Xenocs SA) (Li *et al.*, 2008 ▸), serves as the only guard slits. For a coherent and small-beam setup, the collimation is obtained by slits P and S_4_, and slits S_3_ and S_4_ are set to similar values.

### 2.3. Flight tube and detectors

The detector flight tube of length 34 m and diameter 2 m is constructed using 8 mm thick stainless steel (Added Value Solutions, AVS). It houses the different detectors and at the same time provides a radiation protection shield without a lead hutch. Three detectors for SAXS, USAXS and XPCS are enclosed inside a motorized wagon at ambient pressure, and the carriage travels precisely along a rail system within the tube, enabling very accurate automated change of the sample-to-detector distance between 0.8 and 30.8 m (Van Vaerenbergh *et al.*, 2016 ▸). The front flange of the wagon has an X-ray transparent fibrous carbon window (PEP Compositec), of thickness ∼400 µm and diameter 260 mm, which separates the vacuum of the tube outside and atmospheric pressure inside during normal operation. The flight tube is evacuated to 10^−2^ mbar (1 Pa) by means of an industrial dry screw pump (Edwards GXS450). The temperature inside the wagon (295 K) is regulated by a fan-assisted heat exchanger system. Two independent beamstop systems are installed outside the wagon in front of the vacuum window. The main beamstop (a lead block of height 2 mm, width 5 mm and thickness 2 mm) has an implanted planar silicon photodiode (Advanced Photonix Inc.) that serves as a transmitted beam intensity (*I*_T_) monitor. Auxiliary beamstops of circular shape with diameters 1, 3, 6 or 12 mm are used for fibre diffraction and XPCS applications. To access the lowest *q* (≤10^−3^ nm^−1^), a rectangular beamstop of size 1 × 3 mm is employed, while grazing-incidence SAXS (GISAXS) measurements make use of a lead strip of width 3 mm.

**Figure 4:**
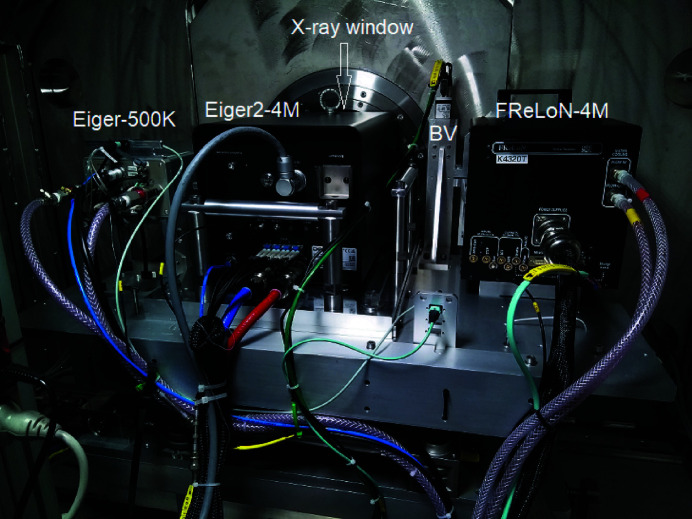
A rear view of the different detectors installed inside the wagon. The desired detector is selected by translating the table to bring it in front of the fibrous carbon window. The detector can also be displaced vertically within the range defined by the window.

Fig. 4 ▸ shows the different detectors installed on the motorized table (*XZ*) inside the wagon behind the fibrous carbon X-ray window. The principal SAXS/USAXS detector is an Eiger2 4M (Dectris AG) hybrid pixel-array detector that replaced the previously used Rayonix MX170 HS (Narayanan *et al.*, 2018 ▸). This detector can also be employed for slower XPCS measurements, while the main XPCS detector is an Eiger 500K (Paul Scherrer Institute version) (Dinapoli *et al.*, 2011 ▸). Both Eiger detectors have a silicon sensor of thickness 450 µm, corresponding to an efficiency of better than 70% at 12.4 keV. The FReLoN 4M fibre-optically coupled CCD detector is mainly utilized for high-resolution fibre diffraction measurements. The beam viewer (BV) is used for beam diagnostics and recording the direct beam at different sample-to-detector distances. The Rayonix LX170 HS WAXS detector is deployed outside the detector tube. All detectors are hardware synchronized with the data acquisition via a time-frame generator as previously described (Narayanan *et al.*, 2018 ▸), and a desired combination can be selected for simultaneous image acquisition.

The main characteristics of these detectors are listed in Table 1 ▸. The spatial resolution tabulated corresponds to the Gaussian FWHM point-spread function (PSF), which was measured by means of an attenuated beam of 20 µm cross section centred on the pixel. The apparent beam profile was fitted to a Gaussian function convoluted with the actual beam profile recorded by the BV. A comparison between the beam profile measured by the Eiger2 4M detector and the BV is shown in Fig. S1 of the supporting information. The spatial homogeneity (flat field) of the SAXS/USAXS/XPCS detectors is regularly calibrated using fluorescence from a 10% HBr (emission at 11.92 keV) solution in a 3 mm diameter capillary illuminated by 13.5 keV X-ray energy. For Eiger detectors, up to 10^6^ counts per pixel were accumulated to obtain good statistical accuracy. After flat-field normalization, the absolute efficiency was determined using water (Milli-Q) scattering in the compressibility limit (∼1.6 × 10^−3^ mm^−1^ at 298 K). With the Eiger2 4M detector, a given sample-to-detector distance typically covers two orders of magnitude in *q* using the standard beamstop, and the smallest 1 mm beamstop extends this range to a factor of about 200.

## 3. Enabling accessories

This section describes the main developments in terms of facilitating advanced experiments using the new source and beamline instrumentation. An essential step is on-the-fly processing and visualization of the treated data, which enables more judicious decision making and fine-tuning of the control parameters during the course of a time-resolved or scanning SAXS or XPCS experiment. Most scientific applications also depend critically on suitable sample environments, which allow the required manipulation of a specimen *in situ*.

### 3.1. Detector data acquisition

All detectors are integrated into the beamline control software, *SPEC* (Certified Scientific Software), via *LIMA* (*Library for Image Acquisition*) device servers. The *LIMA* framework developed at the ESRF provides a unified control interface for 2D detectors. It adopts the concept of image generation and implements common processing tasks for feature extraction and file saving (Petitdemange *et al.*, 2018 ▸). *LIMA* consists of a core library and a multitude of hardware plugins, which are built on top of the software development kit (SDK), providing direct access to detector control and data readout. Strongly optimized for high-performance imaging applications, the *LIMA* servers usually run on dedicated detector control back-ends with essential processing and input/output capabilities. In particular, the servers are equipped with high-speed links to data reduction computers and General Parallel File System (GPFS) servers whenever possible.

The configuration of a 2D data acquisition includes low-level detector parameters like hardware trigger type and number of frames, as well as image processing parameters like background and flat-field correction maps, and the maximum sub-exposure time in software frame accumulation mode. Hardware-accelerated image processing is used when available from the detector, such as pixel binning, vertical/horizontal flip and region of interest. Detector-specific parameters like sensor bit depth or photon-counting threshold energy are also accessible to the user.

In the case of the Eiger2 4M, the SDK is based on SIMPLON (Dectris AG), a representational state transfer (REST)-like application programming interface (API) provided by a dedicated computer, the detector control unit (DCU). The integration of the Eiger2 detectors at the ESRF includes a fibre-optic coarse wavelength division multiplexing/demultiplexing (MUX and DEMUX, respectively) set, inserted between the detector head and the DCU. The MUX integrates the 10 gigabit fibre-optic (FO) links from the detector into a single-mode FO and the DEMUX splits the single link back into multiple FOs to the DCU. This configuration allows the installation of the DCU at the central data storage facility, hundreds of metres away from the beamline, simplifying maintenance and providing high-speed 100 gigabit ethernet connectivity to the *LIMA* server. The *LIMA* Eiger plugin supports both the Stream and FileWriter SIMPLON data retrieval mechanisms; the former is normally used in order to inject the frames rapidly into the image processing chain.

*LIMA* features a multi-threaded hardware image reconstruction interface and this is used for efficient decompression of the frames from the zero message queue stream at the maximum detector data rate listed in Table 1 ▸. The data acquisition hardware is presently optimized for burst modes of 2000 frames at a rate of 1 kHz with the Eiger2 4M and 20 000 frames at a rate of 23 kHz with the Eiger 500K.

### 3.2. Online data reduction

In order to handle the high data rate from these fast detectors efficiently, a new data reduction pipeline based on *Python Fast Azimuthal Integration* (*PyFAI*) (Kieffer & Karkoulis, 2013 ▸) has been developed. Through a graphical user interface (GUI) accessible via *SPEC*, different correction steps can be activated. Depending on the type of detector, main corrections involve dark-image subtraction, flat-field division, spatial distortion correction, unwarping or mapping to reciprocal-space planes *etc*. Corrected 2D patterns are normalized to an absolute intensity scale after taking into account the solid angle subtended by the pixels, detector efficiency, and incident and transmitted beam intensities (Boesecke, 2007 ▸; Pauw, 2013 ▸). All raw and corresponding normalized 2D patterns are saved in hierarchical data format 5 (HDF5) following the NeXus standard (Könnecke *et al.*, 2015 ▸). Once the data acquisition is initiated from *SPEC*, the raw images of one or several active detectors are saved by the corresponding *LIMA* device servers in HDF5 containers. For an acquisition involving a series of patterns, as in time-resolved SAXS (TR-SAXS) or XPCS, the entire data set is saved in a single file.

**Figure 5:**
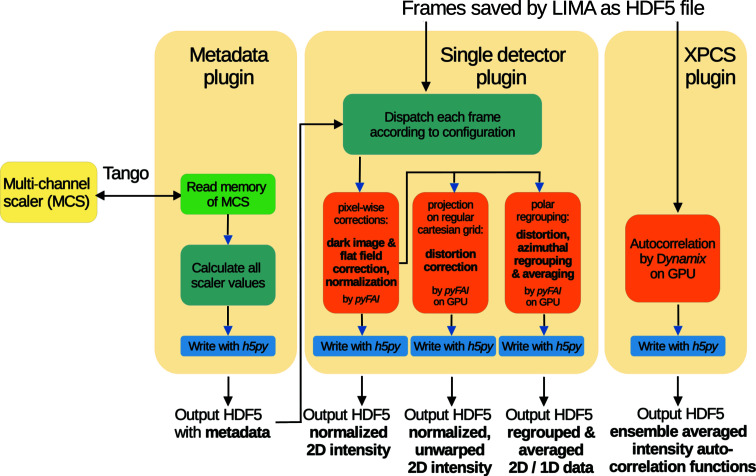
The work flows involved in the data reduction pipelines for SAXS and XPCS. The MCS unit is presently the C216 compact PCI module.

The online data reduction pipeline, *Dahu* (Kieffer & Drnec, 2021 ▸), takes care of gathering the metadata and image data from *LIMA* and multi-channel scaler (MCS) counts, and the processing thereafter. *Dahu* is a lightweight plugin-based Javascript object notation (JSON) remote procedure call (RPC) server framework over *Tango*, which is in use on several beamlines at the ESRF (Götz *et al.*, 2015 ▸). Plugins are written in Python and loaded dynamically. Each job controls one plugin that is executed asynchronously within its own thread, making use of the multiple processors of the system. In this way, the plugin for data reduction can simultaneously process several data sets, composed of hundreds of individual frames, each data set coming from a different detector (usually the SAXS and WAXS detectors). This processing runs while acquisition continues in parallel via *SPEC*/*LIMA*, and thus the reduction step needs to be compatible with the acquisition speed. The reduction plugin, performing intensity correction, image warping and azimuthal regrouping, is based on *pyFAI* for the numerical part and uses the graphics processing unit (GPU) to reach the required performance. Normalized 2D patterns are masked, azimuthally regrouped and averaged to obtain the 1D SAXS profiles, which are saved in individual HDF5 files. For high-throughput operation, the whole sequence is performed in one step as depicted in Fig. 5 ▸, and saving of normalized and regrouped images is optional.

Required parameters for correction are automatically retrieved from the metadata of the recorded frames or from input parameters transmitted as JSON dictionaries from *SPEC*. When reprocessing is required (*e.g.* remasking or refinement of beam coordinates), these jobs can also be run offline using a command line tool or the *SAXSutilities2* program (Sztucki, 2021 ▸). Unlike the online data analysis server, these processing tools are currently not multi-threaded and plugins are run directly for data re-processing. Further plugins are available for the reduction of multispeckle XPCS data based on the Python package *Dynamix* (Paleo *et al.*, 2021 ▸). Beyond the gain in speed when reducing large data sets from high time resolution and high-throughput measurements, the new online data reduction pipeline also features error propagation and *q* binning in nonlinear steps.

### 3.3. Data visualization

**Figure 6:**
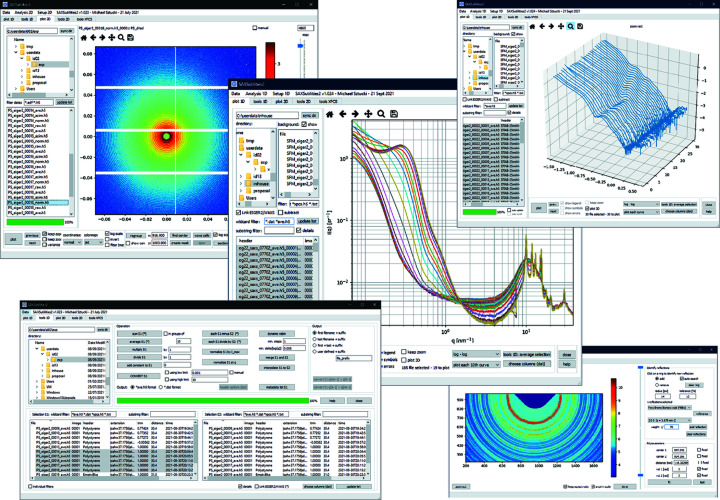
Different features of the *SAXSutilities2* software, such as display of 2D patterns, combined SAXS/WAXS 1D profiles, 3D plots of 1D profiles, and tools for 1D and 2D data treatment. Further details can be found on the program web site (Sztucki, 2021 ▸).

The 1D and 2D data outputs from the data reduction pipeline can be visualized using the *SAXSutilities2* software, which has been rewritten in Python (Sztucki, 2021 ▸). This software is available as a complete binary package (based on *pyinstaller*; https://github.com/pyinstaller/pyinstaller) con­tain­ing all required libraries for Windows and some Linux distributions. This package features the visualization of 2D images in the ESRF data format (EDF) and HDF5 files (as implemented at the ESRF) in different reference coordinate systems (Boesecke, 2007 ▸), finding the beam centre, SAXS/WAXS distance calibration using different calibration standards (such as parabromobenzoic acid, silver behenate, alumina and silicon), and creation of software masks for unusable regions in the scattering patterns. In addition, 2D image operations like averaging, subtraction, and conversion between EDF and HDF5 files are available. A specific interface allows offline data reduction based on the *Dahu* plugin and older *Saxs* programs package (Boesecke, 2007 ▸). The latter also allows averaging of *I*(*q*) over a restricted azimuthal angular range for the analysis of oriented samples. One of the main applications of *SAXSutilities2* is for displaying multiple 1D data sets, optionally after background subtraction, in different plot modes used in small-angle scattering. Data sets can be easily sorted and selected by their file name or metadata (like sample description or sample-to-detector distance) saved in the header of all files. The 1D tool feature of the program includes, among others, averaging, background subtraction, rebinning of the *q* scale, merging of data sets recorded at different sample-to-detector distances, conversion between HDF5 and ASCII data types, and creation of 2D maps of a large set of 1D data from spatial scans. In addition, the intensity autocorrelation functions calculated by the *Dynamix* program can be visualized. Selected features of the program are displayed in Fig. 6 ▸.

The gap between the modules of the Eiger2 4M detector is a nuisance when investigating oriented samples, notably in fibre diffraction. The missing data along an azimuthal sector appear as slots in the corresponding averaged scattering profile. One way to patch these gaps is by making a second acquisition after displacing the detector along the diagonal. This approach is not convenient in time-resolved investigations or for radiation-sensitive samples. Therefore, a feature has been added to rotate the 2D scattering pattern by 180° with respect to the beam centre and patch the gaps, as illustrated in Fig. S2. This procedure still leaves some holes at the intersection of horizontal and vertical gaps, which can be further patched by mirroring the image.

### 3.4. Sample environments

Advances in beamline instrumentation were further complemented by the refurbishment of some of the existing sample environments, which include a new rapid stopped-flow mixing device (SFM 4000/MS 70, Biologic), a new Mettler–Toledo heating stage (HS82) for temperature ramps, a new piezo actuator (Piezosystem Jena) for the pressure jump setup (Möller *et al.*, 2016 ▸) and Peltier heating/cooling stages for multiple capillaries. In order to facilitate low-background USAXS and UA-XPCS under shear, a rheo-SAXS setup consisting of two concentric quartz capillaries was developed (Narayanan *et al.*, 2020 ▸). The capillaries have a wall thickness of 0.05 mm, the inner capillary has an outer diameter of 1 mm and the outer capillary has an inner diameter of 2 mm. The inner capillary is coupled to the shaft of a stress-controlled rheometer (Haake RS6000). This configuration is a compromise in terms of lower scattering background versus the resolution of rheology due to the small volume (∼50 µl) of the sample involved. Nevertheless, for viscous samples reliable rheological parameters can be derived. The lower background from the cell aids in mitigating the undesired heterodyning effect in XPCS.

For automated delivery of liquid samples, a sample changer based on a 28 port Cheminert stream selector (Valco Instruments) and flow-through capillary cell was developed. This system enables the simultaneous loading of 25 samples, remote sample selection and transfer to the flow-through capillary cell (quartz, inner diameter 2 mm, or 1 mm for smaller sample volumes, and wall thickness 0.05 mm). The capillary can be washed and dried in between successive measurements as part of the data acquisition procedure. This capillary cell is placed with a minimum air gap and it is temperature controlled by a Peltier stage (range ∼280–350 K). For the 2 mm capillary cell, the minimum sample volume required is about 25 µl and part of the sample can be retrieved after a measurement.

All sample environments are installed in air at the sample position, with 10–15 µm mica windows (Jahre GmbH) separating the vacuum upstream and the detector tube cone downstream. Air scattering is minimized by means of a telescopic retractable tube arrangement for the upstream vacuum isolation.

## 4. Enhanced performance

This section describes some representative examples of the improved performance of the instrument with the EBS. The most important gains are the less stringent requirement of the collimation, and the consequent increase in flux throughput and reduction in the parasitic background. These factors are beneficial for USAXS and XPCS techniques. Both SAXS/WAXS and USAXS can now be performed with the same collimation setting with maximum flux ∼2 × 10^13^ photons s^−1^. XPCS requires further collimation but still with a flux in excess of 10^12^ photons s^−1^. As a result, very fast dynamics in the ultra-small-angle region can be probed without flux limitations. The earlier onset of radiation damage is a limiting factor when investigating soft matter and biophysical specimens. In this respect, the use of highly efficient photon-counting detectors has become essential for SAXS measurements.

### 4.1. USAXS resolution

**Figure 7:**
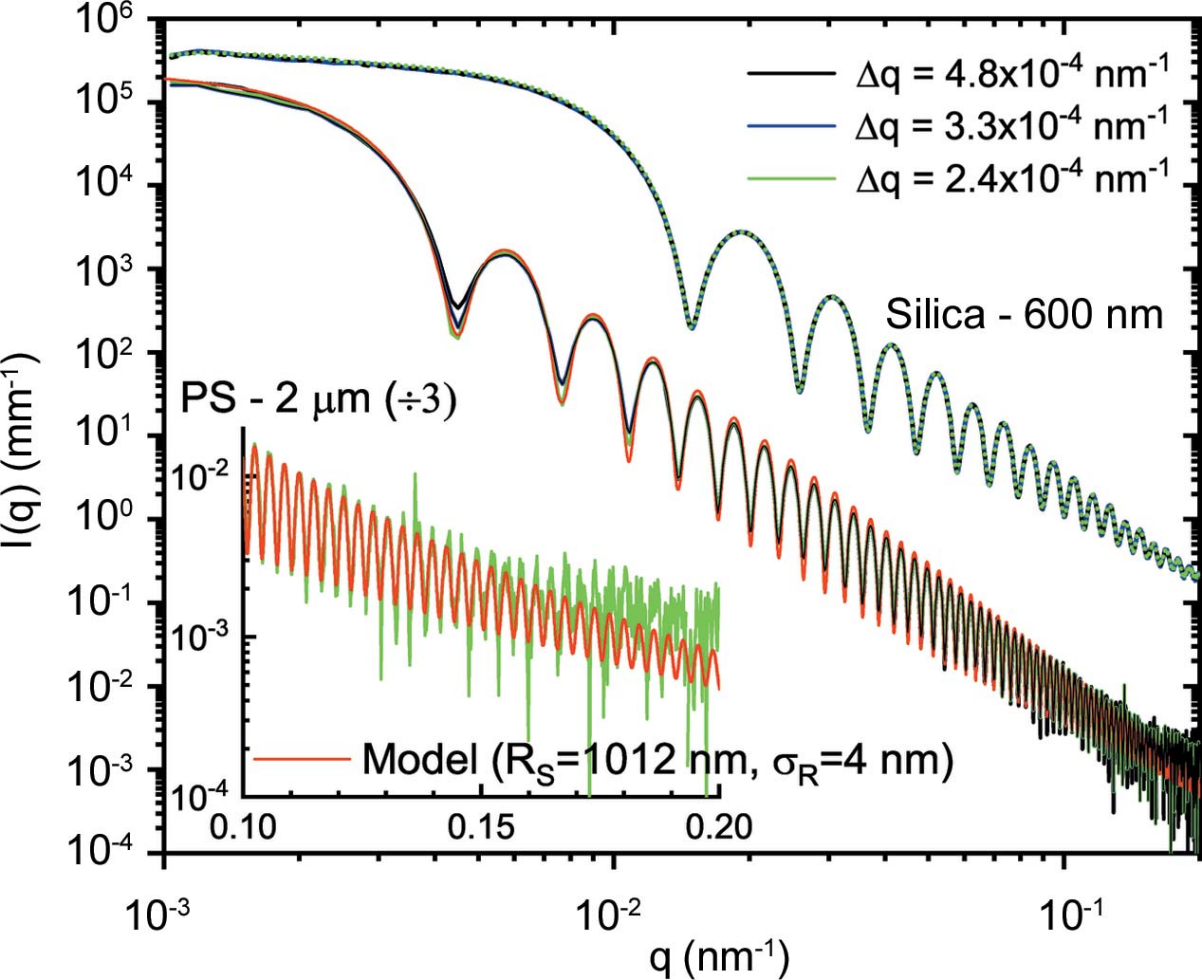
Azimuthally averaged scattering profiles of dilute silica and polystyrene colloidal suspensions recorded with the Eiger2 4M detector at a sample-to-detector distance of 31 m. The mean radii (*R*_s_) of the particles are 300 nm (silica) and 1012 nm (PS) and the corresponding polydispersities in terms of the width of the Gaussian size distribution (σ_*R*_) are 5.4 and 4.0 nm, respectively. The model represents a polydisperse sphere scattering function, which can describe up to the 70th minimum as illustrated in the inset.

The effective *q* resolution (Δ*q*) in the USAXS range with moderate collimation is comparable to the best resolution obtained previously (Narayanan *et al.*, 2018 ▸). Fig. 7 ▸ illustrates the resolution effect roughly corresponding to the collimation conditions presented in Fig. 3 ▸ for two different colloidal suspensions. For smaller particles (Stöber silica) with mean radius *R*_s_ ≃ 300 nm and polydispersity ≃ 1.8%, the resolution effect is not visible since all the profiles superimpose perfectly. However, for larger polystyrene (PS) particles (Polysciences Inc.) with *R*_s_ ≃ 1012 nm and a narrow size distribution, polydispersity ≃ 0.4%, the resolution effect is manifested by the pronounced smearing of the minima in the lower-*q* region. For particles with a narrow size distribution, the smearing due to instrument resolution dominates at low *q* in the absence of multiple scattering (Semeraro *et al.*, 2018 ▸), while the polydispersity influences the most at high *q*, smoothing the oscillations. Therefore, this smearing effect can be used to estimate an average Δ*q* from the required convolution of the polydisperse sphere scattering function to describe the profile. The resulting values are comparable to those estimated from the beam size and detector pixel size. The minimum Δ*q* at 31 m determined by the beam size, beam divergence and detector pixel size (75 µm) is about 2 × 10^−4^ nm^−1^, corresponding to roughly 3 µrad.

As shown in Fig. 3 ▸, the minimum size obtainable on the sample is about 20 µm, restricted by the broadening due to diffraction by the slits. In the vertical direction, the beam is fully coherent and beam broadening emerges for slit sizes below 60 µm. In that case, the minimum size is given by the source size and focusing optics. However, in the horizontal direction the slit collimation works down to a size of about 20 µm. Therefore, with the present optics the transverse coherence length along the horizontal direction is about a factor of 3 lower than that in the vertical. The ultimate value of Δ*q* is determined by the transverse coherence length of the beam and therefore the resolution is superior in the vertical direction (∼1.5 × 10^−4^ nm^−1^).

**Figure 8:**
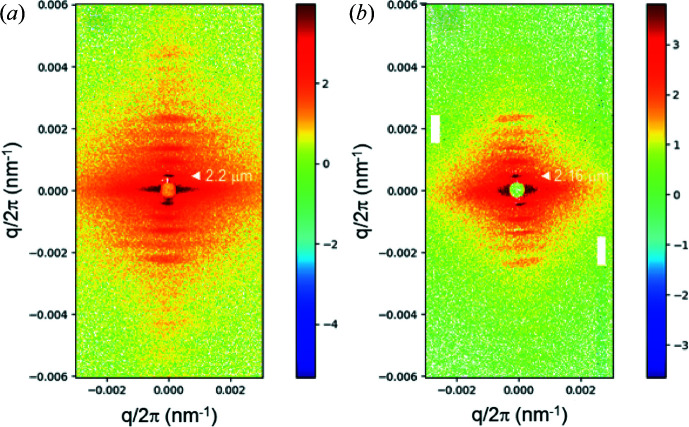
Background-subtracted ultra-low-angle diffraction patterns of a cardiac papillary muscle in the diastole state recorded with 5 ms X-ray exposure. The well defined reflections correspond to the axial repeat of the sarcomeres of length ∼2.2 µm. Patterns (*a*) and (*b*) were recorded by the FReLoN and Eiger2 4M detectors, respectively. The fibre axis is along the vertical and the specimen was provided by courtesy of M. Linari *et al.* (University of Florence, Italy).

The high resolution enables the elucidation of relatively large coherent structures and the probing of their structural dynamics. Fig. 8 ▸ shows ultra-low-angle diffraction patterns from mammalian (rat) cardiac papillary muscle, manifesting the axial repeat of sarcomeres (the unit cell of muscle). The first-order peak corresponds to a sarcomere length of ∼2.2 µm and the higher orders are modulated by an interference function. Usually, the cardiac muscle is much less ordered than skeletal muscle, but a smaller beam cross section (30 × 100 µm) allows one to find coherently diffracting regions and obtain good quality diffraction patterns. In this case, for diffraction patterns recorded in the same preparation and flux (∼10^11^ photons), the FReLoN detector (with 8 pixels binned horizontally) yielded a more resolved diagram. This shows that for muscle fibre diffraction work, CCD-based detectors are still competitive (Reconditi *et al.*, 2017 ▸). The Eiger2 4M pattern was rotated and the gaps between the modules were patched, as mentioned before (Fig. S2). This detector has a major advantage when it comes to time-resolved studies such as probing the dynamics of cardiac muscle regulation (Brunello *et al.*, 2020 ▸).

### 4.2. XPCS performance

**Figure 9:**
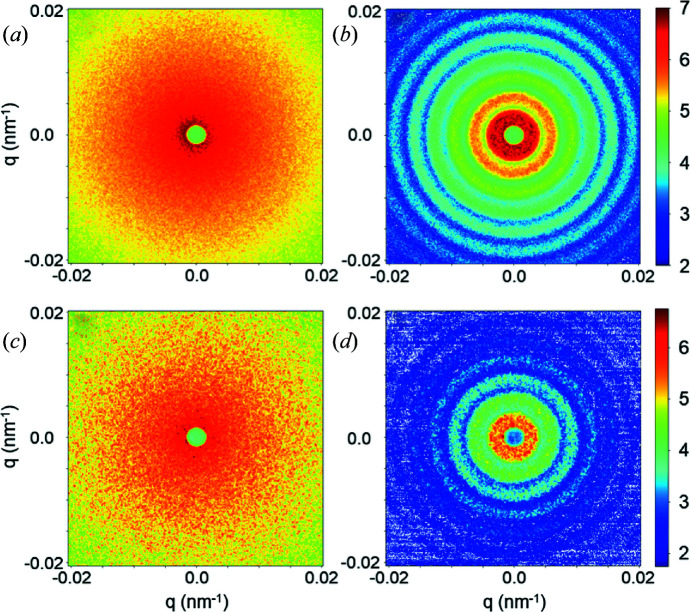
Static speckle patterns from powder samples of (*a*), (*c*) alumina and (*b*), (*d*) dried polystyrene latex particles. The upper and lower panels are registered in the standard USAXS and XPCS modes, respectively, using the FReLoN detector.

The most outstanding property of the EBS and other new-generation synchrotron sources is the orders of magnitude increase in the degree of coherence. This can be exploited for a variety of applications such as XPCS, coherent diffractive imaging *etc.* (Narayanan & Konovalov, 2020 ▸; Lehmkühler *et al.*, 2021 ▸). In this section, the enhanced performance of the XPCS technique in the ultra-small-angle range is illustrated by means of a few representative examples. A fully coherent beam is obtained by closing both slits S_3_ and S_4_ to 40 µm (vertical) and 20 µm (horizontal), resulting in a beam size at the sample of 25 µm (vertical) and 20 µm (horizontal). Fig. 9 ▸ shows the static speckle patterns recorded from dried alumina powder and a film of dried polystyrene particles (the same as in Fig. 7 ▸) in the standard USAXS and XPCS configurations using the FReLoN detector. The contrast of the speckles is significantly better in the latter, as indicated by the fineness of the grains. In static measurements, the speckles make the low-*q* region somewhat noisy, and therefore averaging over time for fluid samples and position for solid samples becomes mandatory.

**Figure 10:**
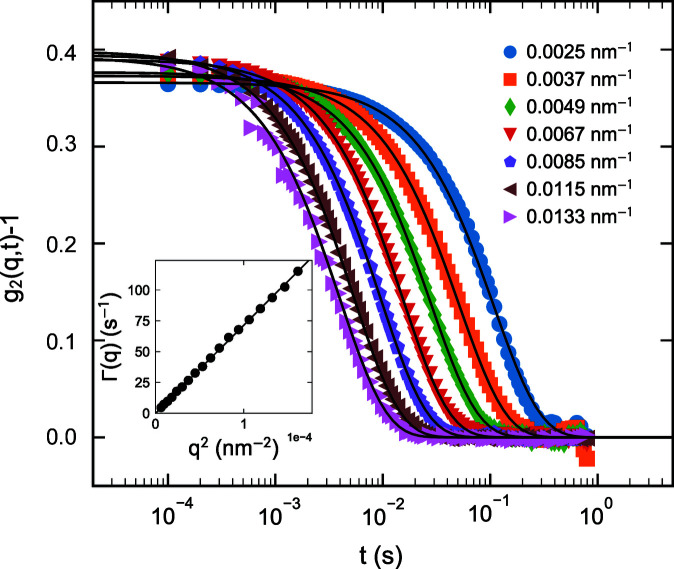
Multispeckle ensemble-averaged intensity–intensity autocorrelation functions [*g*_2_(*q*, *t*)] from a dilute colloidal suspension of silica particles with a mean radius of 300 nm. Continuous lines are exponential fits with relaxation rate Γ(*q*). The inset depicts the *D*_0_ *q* ^2^ behaviour of Γ(*q*) with *D*_0_ ≃ 0.83 µm^2^ s^−1^.

The performance of multispeckle XPCS is illustrated using the same silica colloidal suspension as in Fig. 7 ▸. Measurements were performed using the Eiger 500K detector operating in 8 bit mode and 10 000 frames were acquired in 1 s. Fig. 10 ▸ presents the azimuthally averaged intensity–intensity autocorrelation functions [*g*_2_(*q*, *t*)] as a function of *q*. As expected for Brownian particles, all *g*_2_(*q*, *t*) functions decay exponentially with a relaxation rate Γ(*q*) = *D*_0_ *q* ^2^, where *D*_0_ is the diffusion coefficient. The intercept at *t* = 0 defines the speckle contrast, β, which is close to 0.4, demonstrating the improvement from 0.3 obtained before (Zinn *et al.*, 2018 ▸). Essentially, for XPCS the coherent flux has increased by about a factor of 30 and β improved by 30%. This has already enabled the probing of very fast active motions in dilute suspensions (volume fraction ≃ 0.0003) and direction-dependent analysis of self-propulsion dynamics (Zinn *et al.*, 2022 ▸). As a result, multispeckle UA-XPCS is no longer limited by the detector frame rate or coherent flux.

### 4.3. Time-resolved SAXS

The efficacy of TR-SAXS and TRUSAXS experiments improved primarily due to the high frame rate of the photon-counting detectors, the Eiger2 4M and Eiger 500K. With CCD-based detectors, the readout noise and readout time limited the quality of the data and the efficiency of the measurement. Often, it was necessary to repeat the data acquisition with different starting delays to fill the readout gap in time (Narayanan *et al.*, 2014 ▸). Now it is possible to acquire millisecond frames in a continuous sequence as long as the sample does not manifest radiation damage. In particular, for stopped-flow kinetic studies, the detector frame rate is well matched with the time resolution defined by the dead time of mixing. It turned out that, for many systems, the readout gap during which the tandem fast-beam shutter remained closed was helpful to minimize the radiation damage. As a result, a suitable shuttering scheme (Narayanan *et al.*, 2014 ▸) is still employed for data acquisition rates below 200 Hz, the maximum reliable speed of the shutter.

**Figure 11:**
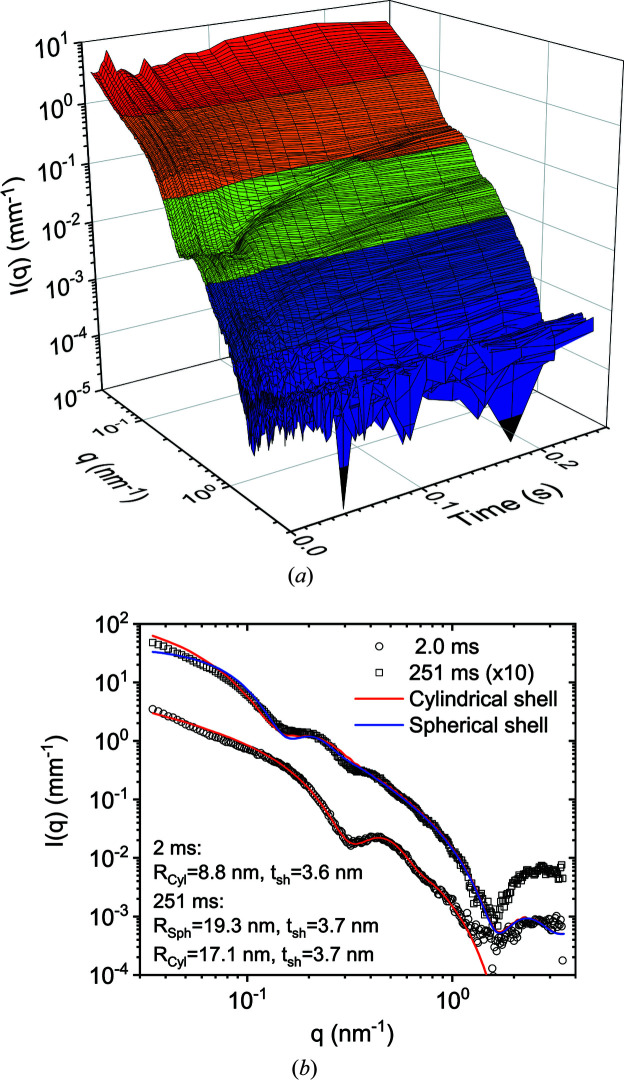
(*a*) Isometric plots of the normalized and background-subtracted SAXS profiles following the rapid mixing of 25 m*M* solutions of cationic TTAB and anionic LPFO surfactants. The scattering features represent mixed unilamellar vesicles, which form spontaneously during the mixing process. Each profile was integrated for 1.8 ms, comparable to the dead time of mixing (*ca* 2 ms), and manifests the growth of unilamellar structures over time. (*b*) Modelling of the initial and final profiles in terms of single-walled tubes and spherical shells with radii *R*_Cyl_ and *R*_Sph_, respectively, and wall or shell thicknesses (*t*_sh_) as shown in the legend.

Fig. 11 ▸(*a*) illustrates the typical quality of SAXS data obtained with millisecond frames involving stopped-flow mixing. In this case, 25 m*M* aqueous solutions of cationic tetra­decyl­tri­methyl­ammonium bromide (TTAB) and anionic lithium per­fluoro­octan­oate (LPFO) surfactants were mixed with a dead time of about 2 ms. Upon mixing, catanionic unilamellar vesicles are expected to form spontaneously, and to grow further over time by a slower ripening process (Weiss *et al.*, 2008 ▸).

The observed behaviour is similar to that previously obtained with a higher concentration (50 m*M*) and a lower time resolution. The quality of the data is superior to that reported in the previous study, which involved averaging three repetitions of three times longer integration and twice the concentration (Weiss *et al.*, 2008 ▸). The minima in the scattering profiles move to lower *q* with time, indicating the growth of these self-assembled structures. Control measurements indicated no change in the scattering profile with the X-ray exposure for this duration (0.25 s), thereby demonstrating the reliability of the results. However, there is an important difference in the shape of the profiles in the low-*q* region, which suggests the formation of more elongated structures than spherical uni­lamellar vesicles. Fig. 11 ▸(*b*) displays the best description of the initial time data obtained with a cylindrical shell structure using the *SasView* program (Doucet *et al.*, 2018 ▸). As their growth progresses, the profile gradually changes towards that typical of spherical vesicles. At 0.25 s, the behaviour is in between spherical and cylindrical shapes and it is likely that the two moieties coexist at that stage. In other words, the nonequilibrium structures initially formed are cylindrical; these structures grow with time and eventually transform to a spherical morphology. The thicknesses of the cylindrical and spherical shells are consistent with a single bilayer of the surfactant mixture and therefore confirm the unilamellar structure. It has now become possible to study even ten times more dilute samples, which show quantitatively different behaviour but will be presented elsewhere.

## 5. Limiting factors

The increased brilliance, detection capability and dynamic range also expose certain limitations, which hamper reaching the ultimate performance. Radiation damage is the most serious issue for the vast majority of samples, and it needs to be identified and circumvented by means of appropriate protocols. With the previous instrument, the SAXS/USAXS intensity dynamic range was often restricted by the digital resolution (16 bit) and average readout noise of the CCD detectors. With photon-counting detectors, this lower threshold has been suppressed, which makes the secondary scattering from the vacuum windows observable.

### 5.1. Radiation damage

Radiation damage is prevalent in almost all soft matter and biophysical studies (Narayanan *et al.*, 2014 ▸; Jeffries *et al.*, 2015 ▸). The onset of damage varies over a large range of X-ray doses, depending on the sample and the prevailing physicochemical conditions. Since samples have to be investigated under variable thermodynamic or physiological conditions, specific solutions need to be adapted for every system. In each experiment, the first step is to assess the threshold of damage by progressively increasing the exposure time (from a few milliseconds) and the interval between successive exposures. The onset of damage is judged on the basis of any systematic change in the scattering profile with increasing X-ray exposure. In standard experiments, the focusing and collimation slits are adjusted such that the beam size is minimized on the detector and larger on the sample. When a small beam spot on the sample is required, the flux, exposure time and interval between exposures are optimized to remain below the damage threshold. In static measurements, the specimen is often translated between successive exposures by a distance greater than the size of the beam. For fluid samples, this is usually achieved by pushing the column within a flow-through capillary cell. In addition, the solutions need to be thoroughly degassed. In time-resolved and XPCS experiments, the sample translation schemes can have inadvertent consequences, and therefore the flux and maximum acquisition times are adapted to remain below the threshold of radiation damage. As a result, the measurements need to be repeated several times with different timing sequences for longer acquisitions. Indeed, for slower time-resolved and XPCS experiments (≤200 Hz), a shuttering scheme is employed. In addition, a factor-10 attenuation is often used to delay the onset of damage and avoid saturation of the detector.

**Figure 12:**
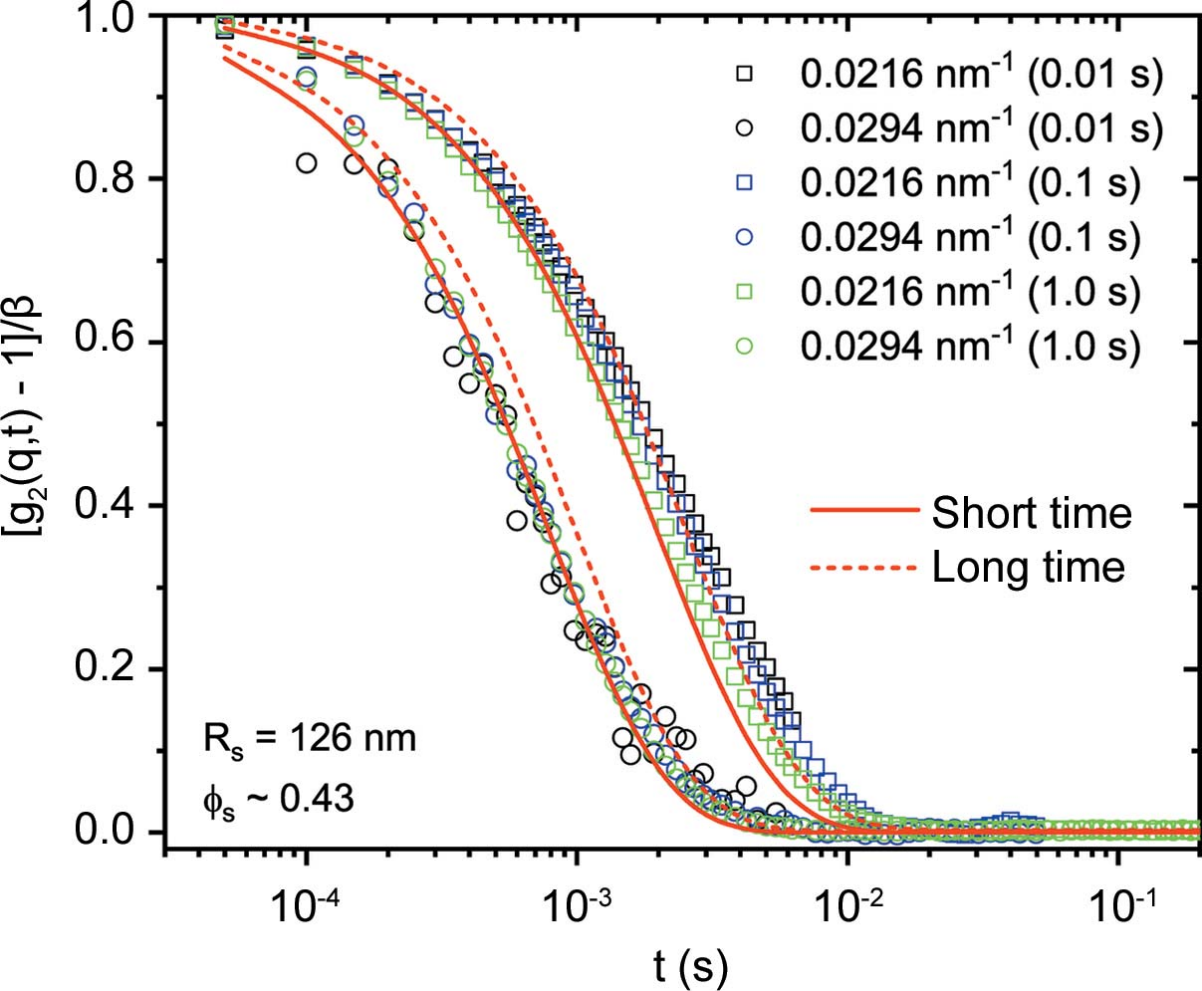
Multispeckle intensity–intensity autocorrelation functions from a concentrated aqueous colloidal suspension of silica particles (mean radius *R*_s_ ≃ 126 nm and volume fraction ϕ_s_ ≃ 0.43) for different acquisition times at the structure factor peak (0.0216 nm^−1^) and above. Continuous and dashed lines are exponential fits to the initial and final decays of *g*_2_(*q*, *t*), respectively.

Fig. 12 ▸ shows that meaningful *g*_2_(*q*, *t*) functions can be constructed with very short exposure times: *ca* 10 ms for a concentrated suspension of silica particles (Stöber) (ϕ_s_ ≃ 0.43). Unlike the dilute sample shown in Fig. 10 ▸, here *g*_2_(*q*, *t*) displays two decays corresponding to the short and long time self-diffusion coefficients *D*_S_ and *D*_L_, respectively (Nägele, 1996 ▸). These two decays can be described by two separate Γ(*q*) and there exists a scaling behaviour (Segre & Pusey, 1996 ▸). In this case, the initial and final Γ(*q*) are related by a factor of 2.5. Additional data corresponding to a larger range of *q* are presented in Fig. S3, where the *g*_2_(*q*, *t*) curves are described by stretched exponential functions involving a mean Γ(*q*).

**Figure 13:**
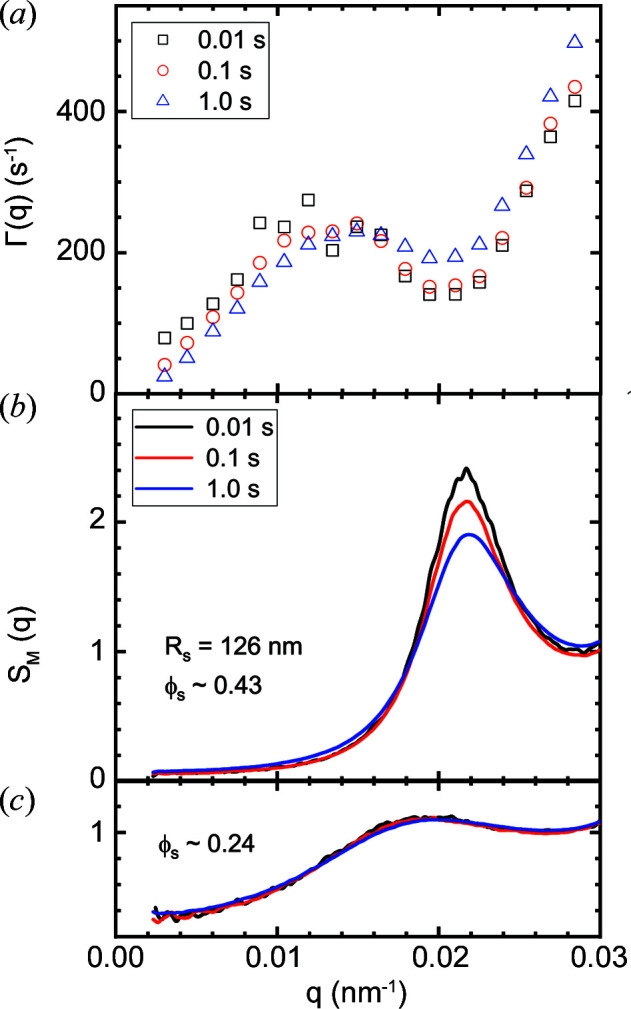
X-ray beam-induced changes in (*a*) the diffusive relaxation rate Γ(*q*) and (*b*) the effective structure factor *S*_M_(*q*) for a concentrated silica particle suspension in the XPCS configuration. (*c*) The same particles at a lower volume fraction (ϕ_s_), showing the much lower influence of the irradiation.

Radiation damage is often detected earlier, and at a greater depth, in static profiles than in dynamics. Fig. 13 ▸ displays an acute case of radiation damage in the XPCS mode with only 6 × 10^11^ photons s^−1^ for a concentrated suspension of silica particles (*R*_s_ ≃ 126 nm and ϕ_s_ ≃ 0.43). The Γ(*q*) data in Fig. 13 ▸(*a*) are derived from the fits in Fig. S3 using a stretched exponential relaxation, and Fig. 13 ▸(*b*) presents the corresponding effective structure factor *S*_M_(*q*) of interparticle interactions (Narayanan, 2014 ▸). The particles are moderately charged and the sample is close to the freezing transition, so if left unperturbed crystalline peaks appear with time. With increasing exposure time, changes in the *S*_M_(*q*) peak are more significant than the variations in the corresponding mean Γ(*q*), thereby demonstrating a greater impact on the local structure than in the dynamics (diffusion). This extreme sensitivity to the X-ray beam is only observed for this range of ϕ_s_, where the sample is close to crystallization. As shown in Fig. 13 ▸(*c*), at lower ϕ_s_ ≃ 0.24 and for dilute samples (presented in Figs. 7 ▸ and 10 ▸) damage is not manifested with several seconds of exposure to the X-ray beam.

The *q* dependence of Γ(*q*) in Fig. 13 ▸(*a*) deviates significantly from the behaviour depicted in Fig. 10 ▸ because of the well known slowing down of dynamics near the peak in *S*_M_(*q*) (Nägele, 1996 ▸). The effect of the beam is to accelerate the dynamics and lower the peak of *S*_M_(*q*), analogous to the addition of salt (Westermeier *et al.*, 2012 ▸). The opposite trend at lower *q* values can be attributed to insufficient averaging with shorter acquisition time as *g*_2_(*q*, *t*) functions were not fully decayed. For the dilute sample as in Fig. 10 ▸, *S*_M_(*q*) ≃ 1, and the scattering form factor and Brownian diffusion co­efficient of the silica particles are not modified by the X-ray beam. Therefore, the ionization effect of the beam is the likely mechanism for the damage in this particular case. A beam-induced speeding up of dynamics has already been noted in oxide glasses (Ruta *et al.*, 2017 ▸). In that case, a semi-empirical approach could be used to estimate the real Γ(*q*) in the zero X-ray dose limit (Chushkin, 2020 ▸).

### 5.2. Limit of intensity dynamic range

**Figure 14:**
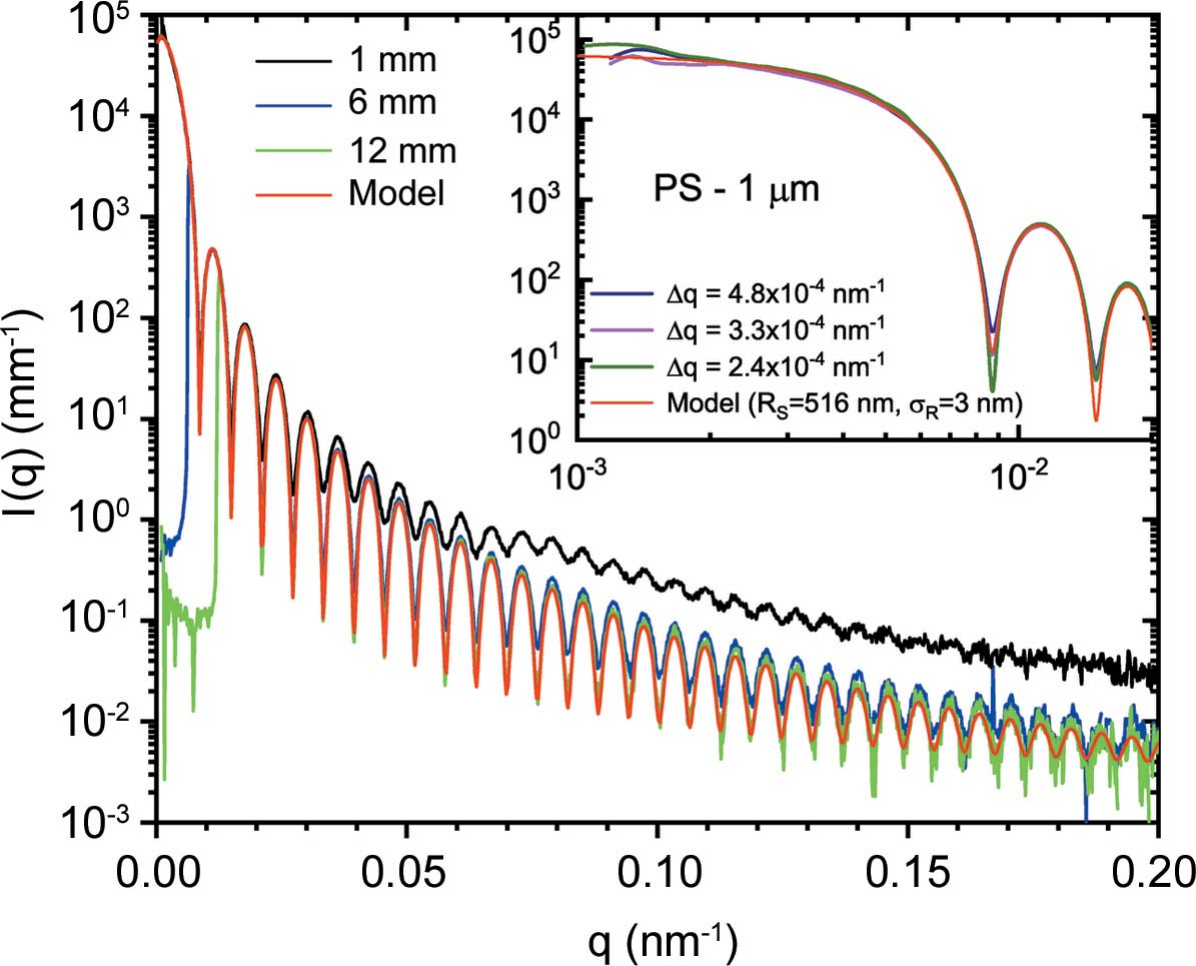
An illustration of the secondary scattering effect using a dilute suspension of polystyrene latex particles of size 1.032 µm, with circular beamstops of different diameters. The effect is very significant for the smallest beamstop of diameter 1 mm. The inset shows the scattering profiles at lower *q* values with different resolutions. Fluctuations at the lowest *q* are caused by the speckles.

With hierarchically organized particulate systems, *I*(*q*) varies over many orders of magnitude. The relatively large active area and high intensity dynamic range of the Eiger2 4M detector offer significant advantages for studying such systems. Previously, using CCD-based detectors, the dynamic range was limited to about 10^5^ by the 16 bit readout electronics. In reality the lower bit is lost by the readout noise, but the azimuthal averaging improves the dynamic range of the reduced scattering profiles. However, when *I*(*q*) decays sharply with *q* as for Porod behaviour (*q* ^−4^), the high intensity at low *q* near the beamstop leads to measurable WAXS from the windows behind the beamstop. In other words, the high intensity at low *q* serves as a secondary source and the resulting WAXS of the window material superimposes on the SAXS and USAXS intensities from the sample. The parasitic WAXS signal generated by the low-angle instrument background can be subtracted out. However, the secondary scattering originating from the strong sample scattering appears as a sample-dependent background which cannot be subtracted out. Furthermore, the standard intensity normalization procedure scales up this secondary scattering contribution due to the small solid angles subtended in USAXS and SAXS. This aspect is illustrated in Fig. 14 ▸ using the USAXS from polystyrene particles (Polysciences Inc.) of size ∼1 µm with a narrow size distribution (polydispersity ≃ 0.6%). Because of the strong scattering from the sample at low *q*, there is an excess intensity in the high-*q* region. As the high intensity at low *q* is successively covered by larger circular beamstops, the scattering profiles approach closer to the model of non-interacting polydisperse spheres. This secondary scattering is a general problem when there is a vacuum window in front of the detector. In this case, it becomes visible below 10^−4^ of the maximum intensity and even more significant below 10^−5^.

This artefact is mainly observed when *I*(*q*) decays steeply and in the presence of sharp oscillations in the high-*q* region of the pattern. For a slower decaying profile with no well defined oscillations, this parasitic contribution will be hidden beneath the actual *I*(*q*). To correct for this excess intensity, it is sufficient to make two measurements with the smallest and largest beamstops. The two profiles can be joined together using the *SAXSutilities2* program to obtain the corrected scattering profile. This correction procedure has already been applied for the data shown in Fig. 7 ▸. At present, the fibrous carbon window separating the atmosphere in the wagon and the vacuum in the flight tube is the main source of secondary scattering. This was independently verified by the measurement of WAXS from the window material and the parasitic profile recorded by the FReLoN detector in the USAXS configuration without a beamstop. The apparent intensity profile measured by the FReLoN superimposes well with the real WAXS when the intensity and *q* scales are recalculated for the actual sample-to-detector distance and solid angle covered by the pixels, as shown in Fig. S4. The effect will be less noticeable when the window is farther from the detector, but the large air gap becomes an issue. Therefore, a compromise was made on the distance between the detector and the window. In general, it is better to install the detector in vacuum without any intervening windows in front (behind the beamstop) if the full dynamic range is to be exploited. This modification will be implemented in a future upgrade.

## 6. Summary

With the EBS upgrade and availability of advanced pixel-array detectors, the ID02 beamline at the ESRF has evolved into a unique multipurpose time-resolved USAXS/SAXS/WAXS (TRUSAXS) and XPCS instrument. For time-resolved studies, the combined *q* range covered is 0.001 ≤ *q* ≤ 50 nm^−1^ using a 1 Å X-ray wavelength. The corresponding nominal size range spans from the micrometre to the ångström scale with sub-millisecond range time resolution. As a result, a wide range of transient processes can be investigated with unprecedented time resolution. The XPCS is more optimized for a *q* range of 0.0015–0.1 nm^−1^, above which the speckle contrast drops significantly.

A *q* resolution down to 2 × 10^−4^ nm^−1^ can be obtained in the standard setup. The improved coherence properties of the beam enable UA-XPCS to be performed, even with relatively dilute samples and probing sub-millisecond dynamics at larger length scales. The high frame rate of the Eiger 500K detector can be exploited for time-resolved XPCS with a time resolution in the second range for the investigation of systems driven out of equilibrium. Efforts are underway to extend XPCS studies to larger biological complexes (Möller *et al.*, 2021 ▸).

The main applications of the instrument are in soft matter and biophysical sciences, but it is also used for a broad range of industrial research and development involving soft mater­ials. Large-scale structural studies of inhomogeneous hard condensed matter systems may also benefit from some of these developments.

## Supplementary Material

Additional figures showing the determination of detector PSF, patching the gaps between detector modules, analysis of XPCS data from a concentrated silica colloidal suspension and the WAXS from fibrous carbon window. DOI: 10.1107/S1600576721012693/jl5029sup1.pdf

## Figures and Tables

**Table 1:** Main specifications of the SAXS, USAXS, WAXS and XPCS detectors for 12.4 keV X-rays The spatial resolution (FWHM) was measured using a 20 µm attenuated beam and fitted to a Gaussian profile. The maximum counts of the CCD detectors are in analogue–digital units (ADU) per pixel. For the Eiger detectors, the PSF is roughly a boxcar function of width the same as the pixel size, and the indicated value is an analogous Gaussian FWHM.

	Eiger2 4M	Rayonix LX170	FReLoN 4M	Eiger 500K
	(SAXS/USAXS)	(WAXS)	(SAXS/USAXS)	(SAXS/XPCS)
Active area (mm × mm)	155 × 163	85 × 170	50 × 50	77 × 39
Pixel size (µm)	75	44.17	23.8	75
FWHM resolution (µm)	52.5 ± 0.5	85.4 ± 1.6	44.3 ± 0.7	52.5 ± 0.5
Dynamic range (photons mm^−2^)	10^9^	10^7^	10^7^	10^9^
Maximum counts	10^7^ s^−1^	65000 ADU	60000 ADU	10^7^ s^−1^
Dark noise	0	<1 photon	<0.2 photon	0
Frame rate (s^−1^)	1000 (8 bit)	8 (2 × 2 bin)	2 (1×1 bin)	23 000 (4 bit)
	500 (16 bit)	100 (10 × 10 bin)	7 (8 × 8 bin)	7000 (12 bit)
