# Supplementary material for: Performance of the time-resolved ultra-small-angle X-ray scattering beamline with the Extremely Brilliant Source
Source: J Appl Crystallogr. 2022 Feb 1;55(Pt 1):98–111. doi: 10.1107/S1600576721012693 (PMC8805168; doi:10.1107/S1600576721012693)
Supplement: Supplementary file 1 [file j-55-00098-sup1.pdf]

JOURNAL OF
APPLIED
CRYSTALLOGRAPHY

Volume 55 (2022)

Supporting information for article:

Performance of the time-resolved ultra-small-angle X-ray scattering beamline with the Extremely Brilliant Source

Theyencheri Narayanan, Michael Sztucki, Thomas Zinn, Jérôme Kieffer, Alejandro Homs-Puron, Jacques Gorini, Pierre Van Vaerenbergh and Peter Boesecke

Performance of the Time-Resolved Ultra-Small-Angle X-Ray Scattering Beamline with the Extremely Brilliant Source

Theyencheri Narayanan,* Michael Sztucki, Thomas Zinn,

Jérôme Kieffer, Alejandro Homs-Puron, Jacques Gorini, Pierre Van Vaerenbergh and Peter Boesecke

ESRF - The European Synchrotron, 38043 Grenoble, France.

E-mail: narayan@esrf.fr

SI. Supporting Information

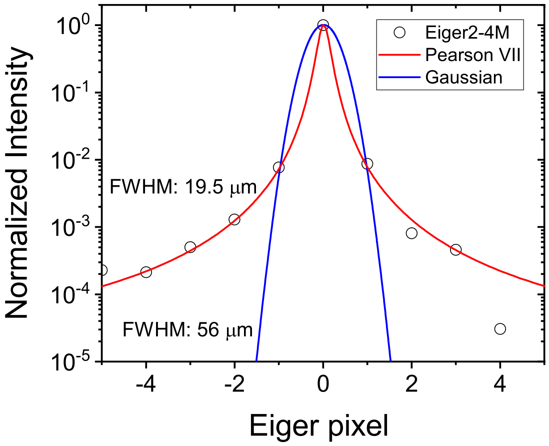

Fig. S1. An example of the measurement of the point spread function of the Eiger2 4M detector using an attenuated beam of 20 μm cross-section centered on the pixel. The actual beam profile recorded by the beam viewer fitted with a Pearson VII function is also shown. For Eiger detectors, the PSF is roughly a boxcar function of width 75 μm, which is approximated as a Gaussian function with FWHM smaller than the pixel size

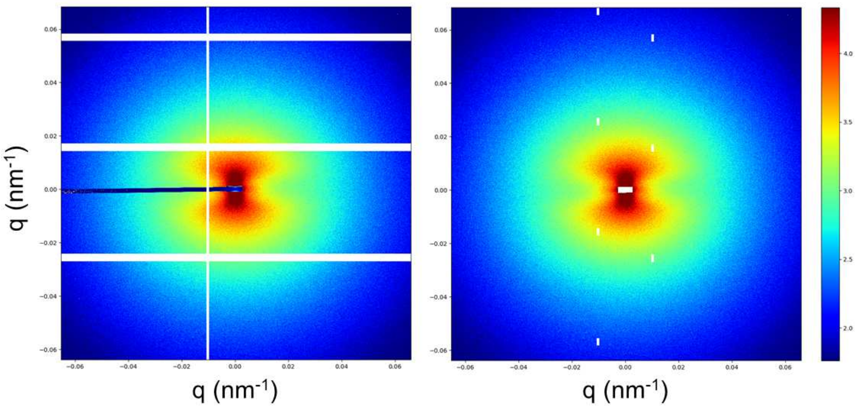

Fig. S2. An example for the patching of the gaps between modules of the Eiger2 4M detector. The left panel shows the normalized USAXS pattern of a deformed elastomer with nanofiller particles. The right panel displays the corresponding patched image after rotating the pattern by 180° with respect to the beam center.

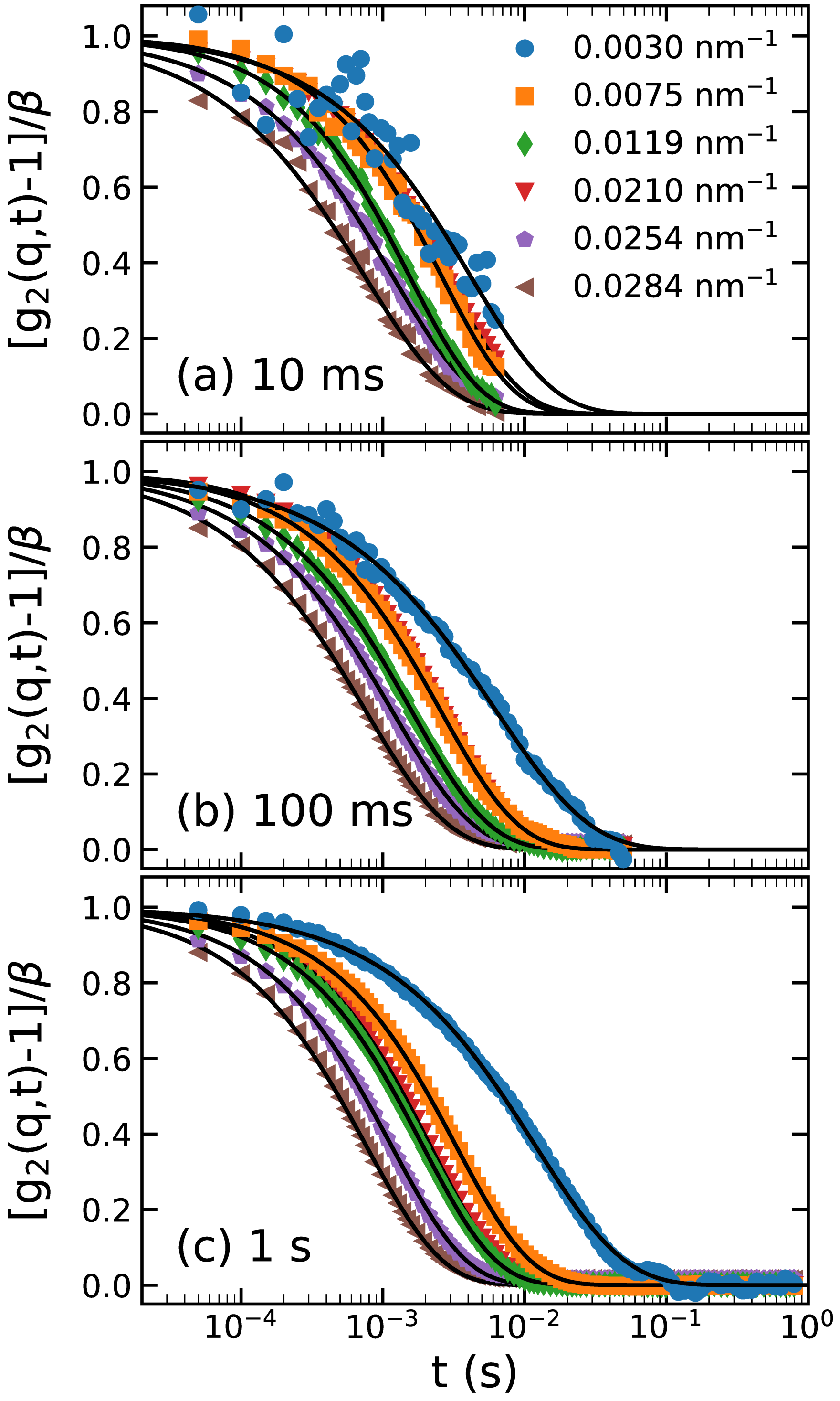

Fig. S3. Multispeckle intensity-intensity autocorrelation functions from a concentrated aqueous colloidal suspension of silica particles (mean radius, $R_s \sim 126$ nm and volume fraction, $\phi_s \sim 0.43$) for different acquisition times as a function of q. Continuous lines are stretched exponential fits with an exponent ~ 0.85.

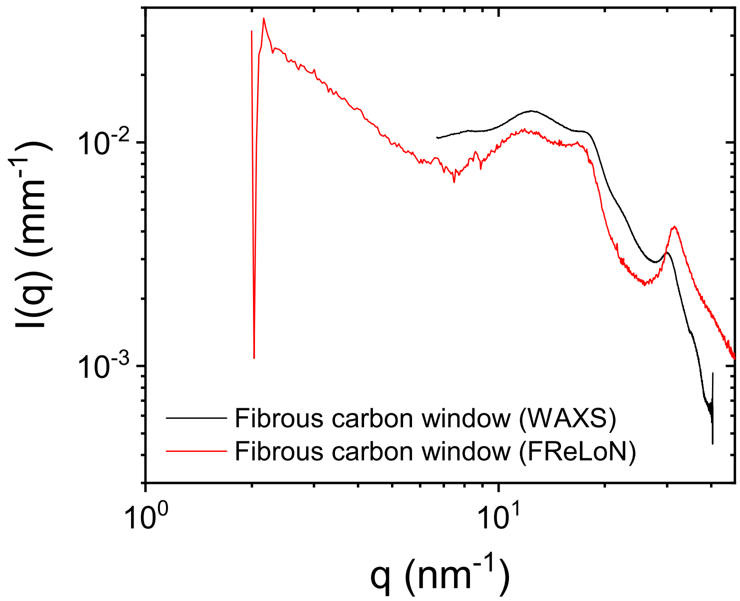

Fig. S4. Comparison of the 1D WAXS profiles from the fibrous carbon window material and that measured with the FReLoN detector without the regular beam stop (i.e. direct beam passing through the fibrous carbon window). For the FReLoN data the q and $I(q)$ scales have been renormalized to the actual sample-detector distance and the solid angle subtended by the pixels. The remaining difference between the two profiles is due to the orientation of the scattering patterns.
